# Recombinant factor VIIa use in patients presenting with intracranial hemorrhage

**DOI:** 10.1186/2193-1801-3-471

**Published:** 2014-08-27

**Authors:** Natalie Yampolsky, Douglas Stofko, Erol Veznedaroglu, Kenneth Liebman, Mandy J Binning

**Affiliations:** Department of Pharmacy, Capital Health Regional Medical Center, 750 Brunswick Ave, Trenton, NJ 08534 USA; Department of Neurosurgery, Capital Institute for Neurosciences, Trenton, NJ USA

**Keywords:** Recombinant factor VIIa, Intracranial hemorrhage, International Normalized Ratio, Anticoagulation, Neurosurgery, Stroke

## Abstract

Recombinant factor VIIa (rFVIIa) can be used for rapid INR normalization in life-threatening hemorrhage in anticoagulated patients. Dosing is unclear and may carry thromboembolic risks. We reviewed the use of rFVIIa at a comprehensive stroke and cerebrovascular center to evaluate dose effectiveness on INR reduction and thromboembolic complications experienced. The primary endpoint was to review the efficacy of rFVIIa in lowering INR. Secondary endpoints included doses used and adverse effects caused by rFVIIa administration. Forty-one percent of patients presented with a subdural hemorrhage. The mean INR prior to rFVIIa administration was 3.5 (0.9-15) and decreased to 1.13 (0.6-2). The mean dose of rFVIIa given was 73 mcg/kg (±24 mcg/kg). Two patients (3%) experienced a thromboembolic event. Recombinant factor VIIa appears to lower INR without significant thromboembolic complications.

## Background

Intracranial hemorrhage (ICH) is a potentially devastating complication of anticoagulation therapy with a mortality rate approaching 50%, twice that of patients not receiving warfarin therapy (Deveras and Kessler 2002; Schlunk et al. 2012). Currently, fresh frozen plasma (FFP) and/or vitamin K are used to reverse warfarin and normalize the international normalized ratios (INR). Unfortunately, the use of FFP requires large administration volumes and both FFP and vitamin K need significant time for reversal lending to be problematic in patients with ICH. Expeditious reversal of coagulapathy with a decrease in time interval to operative intervention may prevent further progression of the bleed and improve transit time to emergency operative procedures (McQuay et al. 2009).

Recombinant factor VIIa (rFVIIa) has been approved by the U.S. Food and Drug Administration for the treatment of bleeding in patients with hemophilia A or B with inhibitors, acquired hemophilia or congenital Factor VII deficiency. Between 2000 and 2008 the off-label use of rFVIIa increased by 143-fold (Lin et al. 2012). Currently, greater than 90% of its use is off-label (Deveras and Kessler 2002; Yank et al. 2011). It is administered at doses that are up to 1000 times the physiologic level, has a half-life of approximately 2.5 hours, and acts by increasing the generation of thrombin on thrombin-activated platelets and through the systemic activation of coagulation (Levi et al. 2010). rFVIIa comes in a 1 mg/mL concentration making it very simple to give large doses, if necessary, in small volumes and in short periods of time. Its effect is also almost instantaneous, making it a viable option for administration in order to facilitate procedural manipulation and possibly reducing the blood loss and the administration of blood products during a procedure. It has been used off-label to treat bleeding associated with a variety of conditions including trauma, surgery, thrombocytopenia, and oral anticoagulant toxicity. This has continued despite the boxed warning of safety concerns with this use.

Although four factor prothrombin complex concentrates (PCC) were approved for reversal of warfarin induced coagulopathy in 2013, it is not widely available at all institutions and in some institutions is more costly than rFVIIa.

There is unclear evidence for the off-label use of rFVIIa, specifically for use in intracranial hemorrhage requiring operative intervention and definitive dosing guidelines have not been established. In addition, efficacy in these settings remains controversial (Freeman et al. 2004; Mayo et al. 2004; Woo et al. 2014). Previous trials have suggested that rFVIIa for off-label purposes may result in an increased rate of thromboembolic events (5.1 to 13.8%) with no added benefit for reduction in mortality rates or improved survival outcomes (Howes et al. 2009; MacLaren et al. 2005; Mayer et al. 2008). The objectives of this study are to review the usage of recombinant factor VIIa for intracranial hemorrhage at a high volume stroke and cerebrovascular center, evaluate the effectiveness of the doses used, and report adverse drug event data in the course of treatment.

## Results

Baseline characteristics of the study are shown in Table 1. A total of 70 patients were identified to have received rFVIIa between January 1, 2010 and June 30, 2013. Twelve patients were excluded from data analyses due to presenting with a non-neurosurgical reason for needing rFVIIa. The majority of patients presented with a subdural hemorrhage (41%) and the average INR prior to rFVIIa administration was 3.5 (0.9-15). The baseline Glascow Coma Scale (GCS) for the study was 8.2 (3-15). Forty-three patients were taking warfarin and one patient was taking dabigatran.

**Table 1:** **Baseline patient characteristics**

Patient characteristics	n = 58
**Mean age (years ± SD)**	67.5 ± 16.325
**Male (%)**	58%
**Mean weight (kg ± SD)**	82.1 ± 22.4
**Anticoagulant at baseline (%)**	79%
**Baseline INR**	3.5 (0.9-15)
**Diagnosis:**	
Subdural hemorrhage	41.4%
Intraparenchymal hemorrhage	24.1%
Subarachnoid hemorrhage	13.7%
Intraventricular hemorrhage	6.8%
Ischemic cerebrovascular accident (Requiring surgical intervention)	8.6%
Other*	5.2%
**Mean GCS** ^**∆**^ **at baseline (min-max)**	8.2 (3-15)

^∆^Glascow Coma Scale.

*Other- thoracic epidural hematoma, emergent lumbar puncture, cervical decompression.

Forty-nine patients (84%) were emergently taken to the operating room after rFVIIa administration, of these, 35 patients (60%) received IV vitamin K and fresh frozen plasma (FFP) in addition to rFVIIa (Table 2). The most common procedure patients received rFVIIa for was a hemicraniectomy (51.7%). An external ventricular drain was placed in 18.9% of the patients, while 15.5% received no surgical intervention (Table 3). The mean dose of rFVIIa given was 73 mcg/kg (±24 mcg/kg) (Table 2). INR decreased to an average of 1.13 (0.6-2) after the first administration of rFVIIa (p < 0.001). Forty-two patients (72%) achieved an INR of 1.4 or less after rVIIa administration. Two patients (3%) experienced an adverse event of thromboembolism that could not be ruled out as secondary to rFVIIa. Both events were deep vein thrombosis (DVT). The average troponin after rFVIIa administration was 0.4173 ng/mL (0.012-10.15) and 4 patients (6.9%) had an increased troponin of greater than 1 ng/mL. The average pH of the patients receiving rFVIIa was 7.39 (7.01-7.56). Seven patients (12%) had a pH of less than or equal to 7.3. Six of the seven patients (85.7%) died (Table 4). Twenty five (43%) total patients died during their hospital admission. Seventeen patients had a cause of death of declining or not improving neurological status, 4 patients died due to respiratory failure, and 4 patients experienced a cardiac arrest.

**Table 2:** **Additional agents used for reversal of INR** ^**§**^

Reversal administration	n = 58
**Received vitamin K only (n, %)**	7, 12%
**Received FFP** ^**∆**^ **only (n, %)**	14, 24%
**Received both vitamin K and FFP (n, %))**	35, 60%

^§^international normalized ratio, ^∆^Fresh Frozed Plasma.

**Table 3:** **Surgical procedure performed**

Procedure	n (%)
**Hemicraniectomy**	30 (51.7)
**External ventricular drain**	11 (18.9)
**No procedure**	9 (15.5)
**Burrholes**	4 (6.9)
**Suboccipital craniectomy**	1 (1.7)
**Lumbar puncture**	1 (1.7)
**t1-t4 laminectomy**	1 (1.7)
**c3-c6 decompression**	1 (1.7)

**Table 4:** **pH and in-hospital mortality**

	Alive	Dead	Hospital mortality
**pH > 7.3 (n = 51)**	32	19	37.2%
**pH ≤ 7.3 (n = 7)**	1	6	85.7%
**Total (n = 58)**	33	25	43%

## Discussion

Our study showed that recombinant factor VIIa is an effective agent to reverse INR in patients presenting in need of a neurosurgical intervention. It also showed that the INR reversal comes with no significant risk of thromboembolic complications. This conclusion is corroborated by other studies that have also investigated the safety of rFVIIa for warfarin patients (Deveras and Kessler 2002; DeLoughery et al. 2013; H-Y et al. 2012). To our knowledge, this is one the largest retrospective reviews looking specifically at the use of rFVIIa administration for patients in need of emergent neurosurgical intervention.

The most effective dose for warfarin reversal has not been established, but clinical trials have published doses ranging from 15–90 mcg/kg (Deveras and Kessler 2002). rFVIIa is known to rapidly normalize INR in warfarin-associated systemic bleeding but whether such INR normalization reflects full reversal of coagulopathy remains controversial (H-Y et al. 2012). Twenty percent of patients in our study received a dose of rFVIIa less than 50 mcg/kg which is considered low dosing of rFVIIa. All patients in the low dose group achieved INR normalization of 1.5 or less. Robbins et al. also showed that lower dosing appeared to be as effective as higher dosing in normalizing the INR (Robbins et al. 2013).

The efficacy of rFVIIa may vary under different physiologic conditions. The activity of rFVIIa has been found to be significantly decreased when pH levels are less than 7.02. One study indicated that the activity of rFVIIa decreased by over 90% at a pH of 7.0. Mamtani et al. found 100% mortality in coagulopathic and severely acidotic patients (pH ≤ 7.02) who had high bleeding rates despite use of rFVIIa (Mamtani et al. 2012). We chose to report not only severe acidosis, but also mild acidosis with a pH ≤ 7.3. Even with the inclusion of patients with only mild acidosis, our mortality rate for those patients was 85.7% compared to 37.2% of patients with no acidosis. This further suggests that the efficacy of rFVIIa could be decreased with increases in acidosis.

Diringer et al. showed that the risk of having an arterial thrombotic event was significantly increased when doses of greater than 80 mcg/kg were used (Diringer et al. 2010). While we did not have any arterial thromboembolic events in our study, one patient that was reported with a deep vein thrombosis (DVT) was given a dose of 100 mcg/kg, whereas the second patient was given two doses; the first at 10 mcg/kg which was followed up by a second 58 mcg/kg dose of rVIIa due of lack of response.

rFVIIa did not appear to enhance the incidence of troponin increases in neurosurgical patients. All patients admitted to the Neurosurgical service receive admission and post-operative evaluation of troponins per protocol in order to not overlook any cardiac changes the patient may be experiencing. Four patients had significantly high troponins greater than 1 ng/mL. Two of the patients were determined to have increased troponins due to a stress response, one was due to sepsis, and the fourth one was lower than the troponin taken before rFVII administration. Neither of the two patients that experienced a venous thromboembolism had an increase in their troponins. We, therefore, could not correlate a higher prevalence of thromboembolic events secondary to elevated troponins.

An analysis of 35 randomized controlled trials in 4468 subjects administered rFVIIa for off-label indications demonstrated a small increase in arterial thromboembolism (5.5% versus 3.5%, p = 0.003), but no difference in venous thromboembolism. Risk factors appear to be advancing age and increasing dose (Levi et al. 2010). It has been stated that the risks of thromboembolic events reported in clinical trials may underestimate the true risk of thromboembolic events with rFVIIa due to the fact that many randomized clinical trials did not actively screen for thromboembolic events (Lin et al. 2012). Patients admitted to the Capital Health Neurosurgical Intensive Care Unit are screened every Tuesday and Thursday for deep vein thrombosis via lower extremity Doppler studies assuring a high probability of detecting any thromboembolic event that may have occurred on patients that received rFVIIa. Additionally, all patients receive DVT prophylaxis with subcutaneous heparin and sequential compression devices up on admission to the ICU. In addition, the DVT rate of 3% in this study is not significantly different than the rate seen in other neurosurgical populations who do not receive rFVIIa (Patel et al. 2013).

While rFVIIa is known to normalize INR rapidly, whether such INR normalization reflects full reversal of coagulapathy remains controversial. Hemostasis in the operating room is not always correlated to the INR reversal, but given the literature reported high mortality rates of approximately 50% in patients with intracerebral bleeding, it remains understandable that clinicians may wish to try potentially helpful intervention despite lack of evidence (Schlunk et al. 2012; Donovan et al. 2012). The mortality rate of our study, 44%, suggests that rFVIIa was only administered to the sickest patients that needed reversal emergently in order to attempt intervention as soon as possible. Severity of illness was determined by the attending neurosurgeon by their evaluation of patients being prone to bleed expansion, location of bleed, general patient presentation, and potential for deterioration. A high severity of illness has an expected 30 day mortality of 72-100% (Greenberg 2010).

The use of PCC has gained some popularity for emergent reversal of anticoagulation (Awad and Cocchio 2013), this use is novel and prior to the approval of 4 factor PCC, our emergent anticoagulant reversal agent was rFVIIa. At our institution, the price for PCC is $1.27/unit with an average dose of 25–50 units/kg. In a 70 kg patient, this equates to $2,200-$4,400. The cost of rFVIIa is $1467/mg with an average dose of 4–6 mg/patient, or $5,868-$8,802. PCCs have only been available at our institution for the past 10 months and since we are a comprehensive stroke center that receives many direct transfer patients from the region’s hospitals, we have come to realize that PCCs are not widely available at all institutions.

The study had several limitations. Although one of the larger studies of rVFIIa use for neurosurgical patients, our overall sample size is small and this limits our power to detect small effects. This is also a retrospective review of rFVIIa use with no control group which can cause systematic biases and potential confounding variables that do not allow for definitive conclusions. The range in doses administered was quite wide and was completely based on the preference of the attending neurosurgeon. In addition, in the future, we plan to compare our results with reversal of INR and outcomes with rFVIIa versus PCCs and the use with the newer anticoagulation medications.

## Conclusion

rFVIIa appears to rapidly and effectly reverse coagulopathy in patients with intracranial hemorrhage who require neurosurgical intervention without an increase in thromboembolic events.

## Methods

This retrospective chart review was performed in accordance with the ethical standards set by the institutional review board. After obtaining institutional review board approval, a retrospective chart review was conducted to identify anti-coagulated patients with intracranial hemorrhage that received rFVIIa. Between January 1, 2010 and June 30, 2013, 58 patients were treated with rVIIa in Capital Health Regional Medical Center and Capital Health Medical Center – Hopewell for a neurosurgical indication. This time frame was selected because prior to January 2010 neurosurgical services were very limited at Capital Health. Patients were excluded if they were younger than 18 years of age or if they needed rFVIIa for a non-neurosurgical indication. All patients receiving rFVIIa during the time period were identified via hospital billing records. A chart review was then conducted on these patients to identify the indication for rFVIIa. At our institution, rFVIIa is restricted for off-label use in Neurosurgery and during Massive Transfusion Protocol only.

The primary objective of this study was to review the efficacy of rFVIIa. Secondary objectives include reviewing doses used and adverse effects caused by rFVIIa administration. Doses of rFVIIa and the administration of subsequent doses were recorded for all patients and converted to a weight based dose using the patient’s actual body weight. The effectiveness of doses used was assessed by the ability of rFVIIa to correct the INR, which was determined by comparing INR after rFVIIa administration to pre-administration INR using a Wilcoxon signed rank test. Administration of vitamin K and FFP were collected for each patient. Adverse drug events were defined as deep vein thrombosis, pulmonary embolism, bowel or limb ischemia, myocardial infarction or ischemic stroke occurring after administration of rFVIIa and throughout the hospital admission. Progress notes, discharge summaries, and imaging studies were evaluated to identify adverse drug events.

## Abbreviations

FFP: Fresh frozen plasma
INR: International normalized ratio
rFVIIa: Recombinant factor VIIa
PCC: Prothrombin complex concentrates
GCS: Glascow coma scale
DVT: Deep vein thrombosis.
